# NOX2 Is Critical to Endocardial to Mesenchymal Transition and Heart Development

**DOI:** 10.1155/2020/1679045

**Published:** 2020-06-16

**Authors:** Hoda Moazzen, Yan Wu, Anish Engineer, Xiangru Lu, Simran Aulakh, Qingping Feng

**Affiliations:** ^1^Department of Physiology and Pharmacology, Schulich School of Medicine and Dentistry, Western University, London, Canada; ^2^Institute of Molecular and Cellular Anatomy, Medical Faculty, Wendlingweg 2 RWTH Aachen University, Aachen, Germany; ^3^Metabolic Syndrome Research Center, Second Xiangya Hospital, Central South University, Hunan, China; ^4^Department of Medicine, Schulich School of Medicine and Dentistry, Western University, London, Canada

## Abstract

NADPH oxidases (NOX) are a major source of reactive oxygen species (ROS) production in the heart. ROS signaling regulates gene expression, cell proliferation, apoptosis, and migration. However, the role of NOX2 in embryonic heart development remains elusive. We hypothesized that deficiency of *Nox2* disrupts endocardial to mesenchymal transition (EndMT) and results in congenital septal and valvular defects. Our data show that 34% of *Nox2^−/−^* neonatal mice had various congenital heart defects (CHDs) including atrial septal defects (ASD), ventricular septal defects (VSD), atrioventricular canal defects (AVCD), and malformation of atrioventricular and aortic valves. Notably, *Nox2^−/−^* embryonic hearts show abnormal development of the endocardial cushion as evidenced by decreased cell proliferation and an increased rate of apoptosis. Additionally, *Nox2* deficiency disrupted EndMT of atrioventricular cushion explants *ex vivo*. Furthermore, treatment with N-acetylcysteine (NAC) to reduce ROS levels in the wild-type endocardial cushion explants decreased the number of cells undergoing EndMT. Importantly, deficiency of *Nox2* was associated with reduced expression of *Gata4*, *Tgfβ2*, *Bmp2*, *Bmp4*, and *Snail1*, which are critical to endocardial cushion and valvoseptal development. We conclude that NOX2 is critical to EndMT, endocardial cushion cell proliferation, and normal embryonic heart development.

## 1. Introduction

Congenital heart defects (CHDs) are birth defects in infants affecting about 1% of live births [1, 2]. The most common forms of CHDs are malformations of septal and valvular structures, accounting for more than 40% of the cases [3, 4]. An intricate network of signaling molecules and transcription factors in the epicardium, myocardium, and endocardium regulates cardiac morphogenesis [5]. While advances in genetic analysis have assisted in identifying genomic factors responsible for morphological abnormalities in patients with CHDs [6], only less than 20% of CHDs are attributed to chromosomal abnormalities or genetic mutations [7, 8]. Over 80% of CHDs have nongenetic or unknown causes that may involve various environmental factors such as maternal pregestational diabetes, obesity, and smoking [9], indicating our limited knowledge on factors that regulate cardiac morphogenesis.

Reactive oxygen species (ROS) are important signaling molecules that modulate the intracellular redox state and gene expression profiles to regulate cell proliferation, differentiation, apoptosis, and migration [10, 11]. An imbalance in ROS production may have adverse effects on fetal development [12, 13]. To this end, we and others have shown that higher ROS levels in the embryonic heart alter gene expression profile and result in a wide range of CHDs, suggesting that overproduction of ROS disturbs normal heart development [14–16].

A critical process in embryonic heart development is the epithelial to mesenchymal transition (EMT). The endocardial EMT (EndMT) is initiated at E9.5 in mice when endocardial cushion swellings are formed in the outflow tract (OFT) and atrioventricular (AV) canal regions [17]. With contributions from neural crest cells, endocardial cushions at OFT and AV canal form the aorticopulmonary septum/semilunar valves and AV valves/cardiac septum, respectively. EndMT is regulated by transcription factors and signaling molecules produced in the adjacent myocardium and endocardial cushions [18].

NADPH oxidases (NOX) are a family of O_2_^−^- and H_2_O_2_-producing enzymes expressed in both phagocytic and nonphagocytic cells [19]. The enzyme complex is composed of NOX proteins (NOX1-5 and DUOX1-2), p22^phox^, p40^phox^, p47^phox^, p67^phox^, and Rac GTPase. ROS production from NOX enzymes by phagocytes plays an important role in killing invading pathogens. The heart also expresses NOX proteins. A major source of intercellular ROS production in adult cardiomyocytes and embryonic cardiac stem cells is NOX2 and NOX4 [20, 21]. Mutations of NOX genes result in chronic granulomatous disease, a rare condition occurring in 1 : 200,000-450,000 live births [22]. Notably, atrial septal defects are also seen in patients with chronic granulomatous disease [23], suggesting that mutations of NOX genes or a lack of NOX-derived ROS production may cause CHDs. However, the role of NOX enzymes in regulating cardiac morphogenesis and their underlying molecular mechanisms are not clear. In this study, we tested the hypothesis that deficiency of *Nox2* disrupts EndMT and results in congenital septal and valvular defects. Our data show that *Nox2^−/−^* mice exhibit cardiac septal defects and valvular abnormalities. Furthermore, deficiency of *Nox2* impairs EndMT and AV endocardial cushion development. Our study reveals a critical role of NOX2-derived ROS signaling in EndMT and normal heart development.

## 2. Materials and Methods

### 2.1. Animals

*Nox2^−/−^* (B6.129S-*Cybb^tm1Din^*/J, Stock No. 002365) and C57BL/6 mice were purchased from Jackson Laboratory (Bar Harbor, Maine). *Nox2^−/−^* mice were backcrossed to C57BL/6 background for more than ten generations; therefore, C57BL/6 mice were used as a control in all experiments. PCR analysis was performed to validate the *Nox2* gene knockout model using the following primers: 5′ AAGAGAAACTCCTCTGCTGTGAA 3′ and 5′ GTTCTAATTCCATCAGAAGCTTATCG 3′, provided by Jackson Laboratory. A breeding program was implemented to harvest fetal and postnatal mice. Animals in this study were handled in accordance with the *Guide for the Care and Use of Laboratory Animals*, published by the U.S. National Institutes of Health (8^th^ edition, 2011). All procedures involving mouse handling and manipulation were in accordance with the guidelines of the Canadian Council of Animal Care and approved by the Animal Care Committee at Western University, Canada.

### 2.2. Heart Morphology and Immunohistochemistry

Heart morphology in postnatal day 0 (P0) mice was analyzed on serial heart sections under a microscope. Briefly, the mouse thorax was fixed in 4% paraformaldehyde overnight, dehydrated in ethanol, embedded in paraffin medium, and sectioned transversely to 5 *μ*m serial sections. For assessment of AV valve thickness and length, serial sections of each heart were evaluated at P0. The longest and thickest region of each valve leaflet was quantified in at least 3 serial sections. E10.5 embryos were harvested to assess cell density in the AV cushion. For immunohistochemical staining, antigen retrieval was performed in citric acid buffer (0.01 M, pH 6.0) for 12 minutes at 94°C using a microwave oven (BP-111, Microwave Research & Applications, Carol Stream, Illinois). Tissue sections were incubated with the following primary antibodies overnight: Ki67 (1 : 500, Abcam), activated caspase-3 (1 : 800, Cell Signaling), Snail1 antibody (1 : 300, Abcam) and NOX2 (1 : 500, BD Transduction Laboratories) followed by one of the following secondary antibodies (Vector Laboratories) for an hour: biotinylated goat anti-rabbit IgG (1 : 500) or biotinylated goat anti-mouse IgG (1 : 500). Signals were amplified by incubation with the ABC reagent (Vector Laboratories) and visualized using 3,3′-diaminobenzidine tetrahydrochloride (Sigma-Aldrich). Heart sections were counterstained with modified Mayer's hematoxylin (Thermo Scientific), and images were captured using a light microscope (Observer D1, Zeiss, Germany).

### 2.3. Analysis of ROS Levels

Frozen sections (10 *μ*m) of E10.5 hearts were employed to assess superoxide levels using dihydroethidium (DHE) (Invitrogen Life Technologies, Burlington, Canada) as we previously described [15]. Briefly, heart sections were incubated with 2 *μ*M DHE for 30 minutes in a humidified and light-protected chamber in room air at 37°C. DHE fluorescence signals were detected using a fluorescence microscope (Observer D1, Zeiss, Germany). Myocardial images (5 from each heart section) were captured using fixed exposure time for both groups. The intensity of fluorescence signals per myocardial area was quantified using AxioVision software.

### 2.4. Real-Time RT-PCR Analysis

Total RNA was extracted from E10.5 fetal hearts using a RNeasy Mini kit (Qiagen, Burlington, ON) as per manufacturer's instructions. cDNA was synthesized using M-MLV reverse transcriptase. At least two hearts were pooled for each qPCR analysis. Real-time PCR was conducted using EvaGreen qPCR MasterMix (Applied Biological Materials, Vancouver, BC). Specific primers were designed for *Nkx2.5*, *Gata4*, *Gata5*, *Tbx5*, *Bmp2*, *Bmp4*, *Tgf-β2*, *Snail1*, and *Mef2c* (Table 1). Samples were amplified for 35 cycles using Eppendorf Realplex (Eppendorf, Hamburg). The mRNA levels in relation to 28S ribosomal RNA were determined using a comparative C_T_ method [15].

### 2.5. *Ex Vivo* Endocardial Cushion Explant Culture

Endocardial to mesenchymal transition (EndMT) was assessed *ex vivo*. AV cushions of similar size E10.5 embryos from *Nox2^−/−^* and control dams were harvested and cultured on collagen gel. Collagen (1 mg/ml, type I collagen of rat's tail, BD Biosciences) was prepared in M199 culture media (M5017, Sigma). Casted collagen was hydrated by OPTI-MEM media plus 1% of fetal bovine serum (FBS) and insulin-transferrin-selenium (ITS) for 30 minutes at 37°C. The AV cushion regions together with the overlying myocardium were explanted, cut open, and seeded with the cushion side facing the collagen gel at 37°C overnight. The following day, the AV cushions adhered to the collagen gel and M199 media with 10% of FBS were added to the explants. To inhibit ROS production, heart explant cultures were treated with 5 mM N-acetylcysteine (NAC). The number of spindle-shaped cell outgrowth from the explanted cushions was quantified 3 days post culturing [24]. Phase contrast images were captured using an Observer D1 microscope (Zeiss, Germany).

### 2.6. Statistical Analysis

Data are presented as means ± SEM. Statistical analysis was performed using Student's *t*-test or two-way analysis of variance (ANOVA) followed by a Bonferroni post hoc test. The survival rate and incidence of congenital malformations were analyzed by a Chi-squared test. A *P* value of less than 0.05 was considered statistically significant.

## 3. Results

### 3.1. Reduced Viability, Litter Size, and Body Weight in *Nox2^−/−^* Neonates

**Figure 1:**
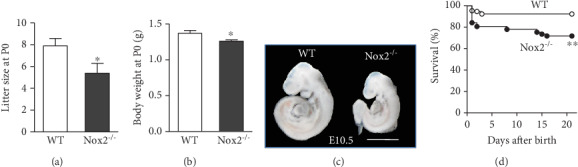
Litter size, body weight, and survival of *Nox2^−/−^* mice. (a) Litter size at birth, calculated based on average number of animals per pregnancy. *N* = 10‐13 litters per group. (b) Body weight of neonates at birth, *N* = 28 samples per group. (c) Representative images of body size of WT and *Nox2*^−/−^ mice at E10.5. Scale bar is 1 mm. (d) Survival rate in *Nox2*^−/−^ mice during the first three weeks of life compared to their age-matched controls. *N* = 129 in the wild-type (WT) group and *N* = 112 in the *Nox2^−/−^* group. ^∗^*P* < 0.05, ^∗∗^*P* < 0.001 by unpaired Student's *t*-test in (a, b) and the Chi-squared test in (d).

Litter size in *Nox2*^−/−^ mice was smaller (*P* < 0.05, Figure 1(a)), and their body weight at birth was significantly lower compared to wild-type (WT) controls (*P* < 0.05, Figure 1(b)). A significant smaller body size or growth retardation was observed in 6 out of 25 (24%) *Nox2^−/−^* embryos collected at E10.5-12.5 while this was not seen in any of the 29 WT embryos (*n* = 4 litters per group, Figure 1(c)). It is possible that the embryos with drastic growth retardation die during gestation, explaining the 25% reduction in litter size at birth. Animal survival after birth was monitered for 21 days with *Nox2^−/−^* mice showing a significant lower survival compared to WT mice (72% vs. 92%, *P* < 0.001, Figure 1(d)).

### 3.2. Septal and Valve Defects in *Nox2^−/−^* Mice

**Figure 2:**
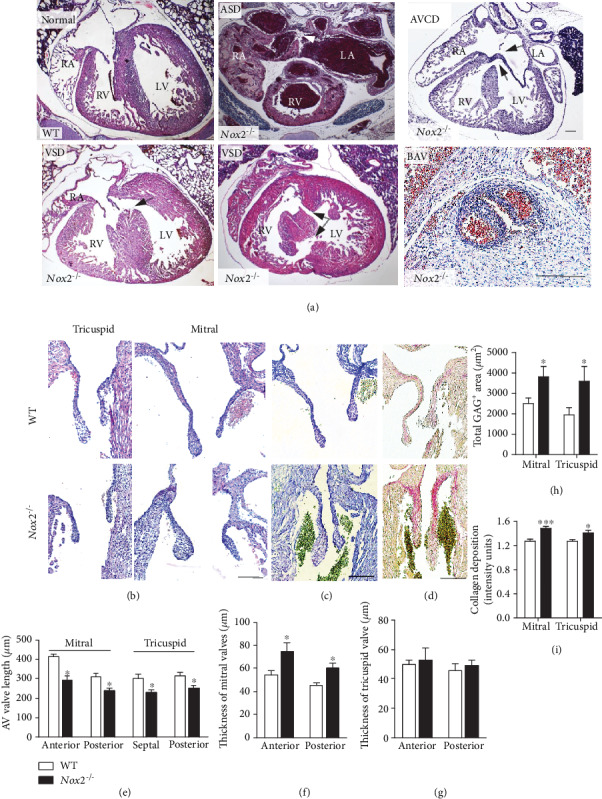
Congenital heart defects in *Nox2*^−/−^ mice at P0. (a) Representative histological images of normal and abnormal hearts. *Nox2*^−/−^ mice exhibited ASD, a complete AVCD (arrows point to interrupted septum primum and VSD), and membranous and muscular (arrows) types of VSD. (b) Representative histological images of tricuspid and mitral valves. (c) Toluidine blue staining of extracellular glycosaminoglycans (light purple) in AV valves. (d) Picrosirius red staining of collagen fibers. (e–g) Quantification of mitral and tricuspid valve length and AV valve thickness. (h) Quantification of the total glycosaminoglycan- (GAG-) positive area in AV valves. (i) Quantification of collagen deposition in mitral and tricuspid valves. ^∗^*P* < 0.05 and ^∗∗∗^*P* < 0.001 vs. WT by Student's *t*-test. *N* = 9‐12 per group. Scale bar in (a) is 200 *μ*m and 100 *μ*m in (b–d).

Histological analysis of *Nox2^−/−^* hearts at P0 shows that 34% of *Nox2^−/−^* mice were born with various CHDs including atrial septal defects (ASD, 18%), ventricular septal defects (VSD, 18%), and severe cases of septal malformation in the form of atrioventricular canal defects (AVCD, 3.3%), which are septation defects (Table 2, Figure 2(a)). Furthermore, 6.6% of *Nox2^−/−^* neonates showed bicuspid aortic valves (BAV, Table 2, Figure 2(a)). Notably, all cases of BAV were associated with septal abnormalities. Most *Nox2^−/−^* mice had a single ASD or VSD. However, 2 out of 61 *Nox2^−/−^* mice (3.3%) had both ASD and VSD. *Nox2^−/−^* hearts with septal defects also had malformations of atrioventricular valves (Figure 2(b)). Specifically, the mitral and tricuspid valves were shortened in length (Figure 2(e)) and the distal tip of mitral (*P* < 0.05) but not tricuspid valves was enlarged in *Nox2^−/−^* mice (Figures 2(f) and 2(g)). In addition, there was a larger area in the AV valves stained by toluidine blue indicating a higher level of extracellular proteoglycan in *Nox2^−/−^* mice (Figures 2(c) and 2(h)). Similarly, picrosirius red staining for extracellular collagen fibers indicated higher levels of collagen deposition in both mitral and tricuspid valves (*P* < 0.001 and *P* < 0.05, Figures 2(d) and 2(i)).

### 3.3. NOX2 Expression Pattern and Endocardial Cushion Formation at E10.5

**Figure 3:**
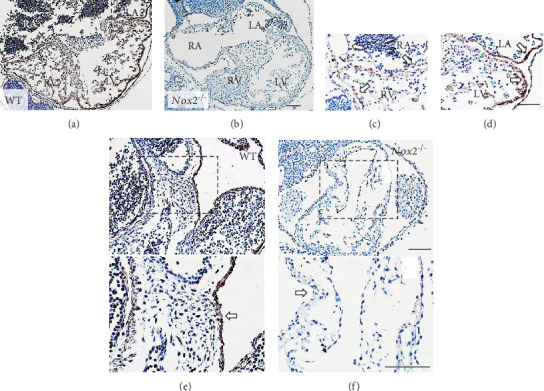
Expression of NOX2 in hearts of E10.5 embryos. (a) Representative immunostaining of NOX2 in WT hearts. (b) Absence of NOX2 staining in *Nox2^−/−^* hearts. (c, d) Enlarged from boxed areas in (a) showing NOX2 expression (arrows) in the atrial and ventricular myocardium of WT hearts. (e) Arrows indicate NOX2 expression in the myocardium overlying AV endocardial cushion in WT but not *Nox2^−/−^* hearts (f). Lower panels of (e) and (f) are enlarged images of the boxed areas in the upper panels. Scale bar is 100 *μ*m.

**Figure 4:**
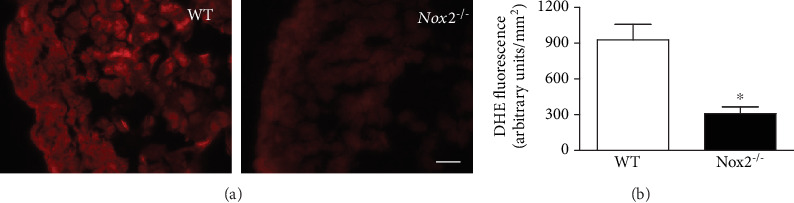
Analysis of superoxide levels in fetal hearts at E10.5 using dihydroethidium (DHE) as a probe. (a) Representative images of ROS levels in the left ventricular myocardium. (b) Quantification of DHE fluorescence intensity. Scale bar is 20 *μ*m. *N* = 3 hearts per group. ^∗^*P* < 0.01 vs. WT by unpaired Student's *t*-test.

To examine the expression pattern of NOX2, immunohistochemical analysis was performed. NOX2 immunostaining was observed in the atrial and ventricular myocardium of WT at E10.5 (Figure 3(a)). NOX2 expression was more robust on the left ventricular myocardium compared to the right (Figures 3(c) and 3(d) ). Importantly, NOX2 was also expressed in the myocardium overlying the endocardial cushions at the AV canal, suggesting a possible role in regulating endocardial cushion development (Figure 3(e)). Notably, the epicardium, endocardium, and cells within the endocardial cushion do not express NOX2 in the WT hearts at E10.5. As expected, *Nox2^−/−^* hearts showed negative NOX2 immunostaining in the myocardium and lower cellular density in the endocardial cushion by hematoxylin staining compared to WT hearts at E10.5 (Figures 3(b) and 3(f)). Furthermore, ROS levels as assessed by dihydroethidium fluorescence intensity were significantly lower in the *Nox2^−/−^* myocardium (*P* < 0.05, Figures 4(a) and 4(b)).

### 3.4. EndMT Is Impaired in *Nox2^−/−^* Hearts

**Figure 5:**
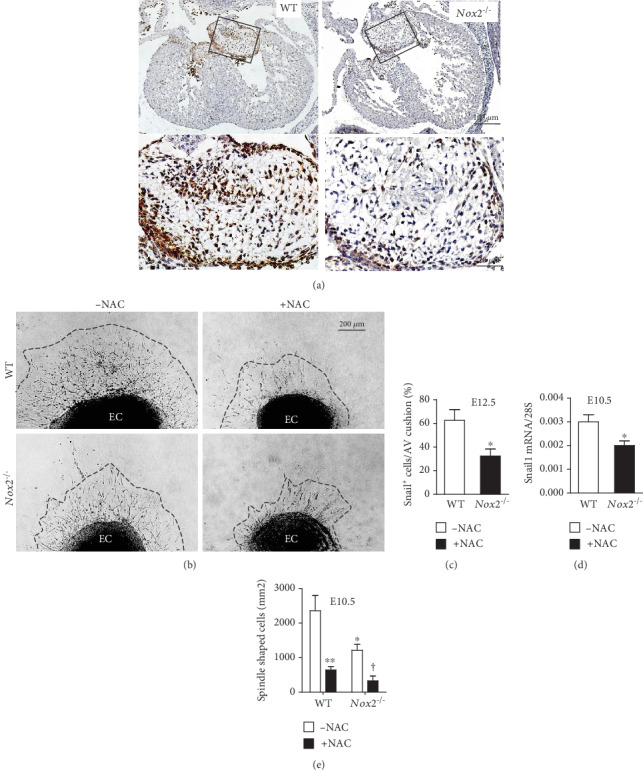
Analysis of endocardial EMT *in vivo* and *in vitro*. (a) Representative images of Snail1 expression in the endocardial cushion (EC) at E12.5. Lower panels are enlarged images of boxed areas in WT and *Nox2^−/−^*, respectively. (b) *Ex vivo* EC culture demonstrates EMT in the presence or absence of N-acetylcysteine (NAC, 5 mM). Dashed line outlines cell migration border. (c) Quantification of Snail1-positive cells in EC at E12.5 (*n* = 4‐5 hearts per group). (d) Analysis of *Snail1* mRNA expression levels in E10.5 full hearts (*n* = 5 hearts per group). (e) Quantification of the number of spindle-shaped cells normalized to explant size (*n* = 3‐5 hearts per group). Data are analyzed by unpaired Student's *t*-test (c, d) and 2-way ANOVA followed by the Bonferroni test (e). ^∗^*P* < 0.05, ^∗∗^*P* < 0.001 vs. untreated WT; ^†^*P* < 0.05 vs. untreated *Nox2^−/−^*. Scale bars are 100 and 20 *μ*m in (a) and 200 *μ*m in (b).

To investigate the role of Nox2 in endocardial cushion formation, we evaluated AV EndMT of endocardial cells *in vivo* and *in vitro*. To this end, expression levels of Snail1, a marker of EMT [25], were analyzed. Our data show that *Snail1* mRNA levels at E10.5 as well as the number of Snail1-positive cells in the AV endocardial cushion at E12.5 were lower in *Nox2^−/−^* compared to WT embryos (*P* < 0.05, Figures 5(a), 5(c), and 5(d) ). To examine EndMT, the AV endocardial cushion of E10.5 fetal hearts was cultured on collagen gel and allowed for cell outgrowth for three days (Figure 5(b)). The number of spindle-shaped cells, which had undergone EndMT, was quantified. *Nox2^−/−^* endocardial cushions had a significantly lower number of spindle-shaped cells compared to WT cushions (*P* < 0.05, Figures 5(b) and 5(e)). To reduce ROS levels, WT and *Nox2^−/−^* AV cushion explants were treated with a ROS quenching agent, N-acetylcysteine (NAC). Notably, treatment with NAC further diminished EndMT of AV cushions in both WT and *Nox2^−/−^* samples (*P* < 0.001, Figures 5(b) and 5(e)).

### 3.5. *Nox2* Deficiency Reduces Expression of Genes Crucial to Cushion Development

**Figure 6:**
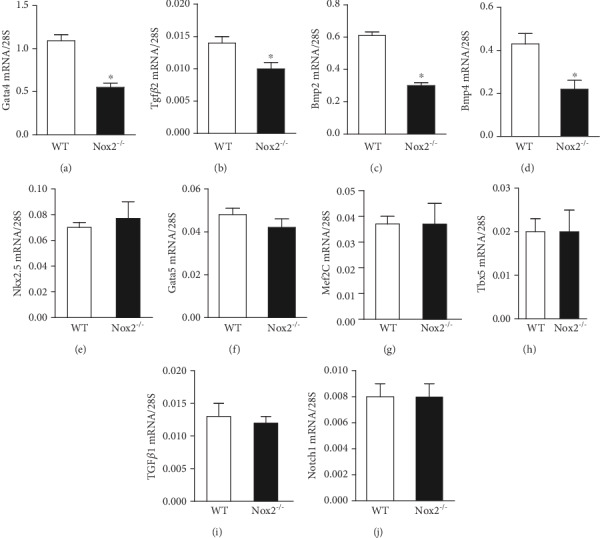
Analysis of mRNA levels in fetal hearts at E10.5. (a–d) The mRNA levels of *Gata4*, *Tgfβ2*, *Bmp2*, and *Bmp4* in *Nox2^−/−^* fetal hearts were significantly lower than those of WT levels. (e–j) Expression levels of *Nkx2.5*, *Gata5*, *Mef2c*, *Tbx5*, *Tgfβ1*, and *Notch1* were not altered in *Nox2^−/−^* fetal hearts. *N* = 7‐9 hearts per group. At least two hearts were pooled for each qPCR analysis. ^∗^*P* < 0.01 vs. WT by unpaired Student's *t*-test.

To further investigate the role of Nox2 in regulating EndMT, we examined the expression of transcription factors and growth factors critical to EndMT and heart development in 10.5 hearts. Our data show that mRNA levels of *Gata4*, a transcription factor important to septal development, were diminished in *Nox2^−/−^* mice (*P* < 0.05, Figure 6(a)). Also, the expression levels of members of the TGF-*β* superfamily, including *Tgf-β2*, *Bmp2*, and *Bmp4*, which are important regulators of endocardial cushion formation [26], were significantly lower in *Nox2^−/−^* fetal hearts at E10.5 (*P* < 0.05, Figures 6(b)–6(d)). We also analyzed mRNA levels of other regulators of cardiac septum formation such as *Nkx2.5*, *Gata5*, *Mef2c*, *Tbx5*, *TGFβ1*, and *Notch1*; however, their expression levels were not significantly altered in *Nox2^−/−^* embryonic hearts (Figures 6(e)–6(j)).

### 3.6. *Nox2* Deficiency Increases Apoptosis and Reduces Cell Proliferation in Endocardial Cushion

**Figure 7:**
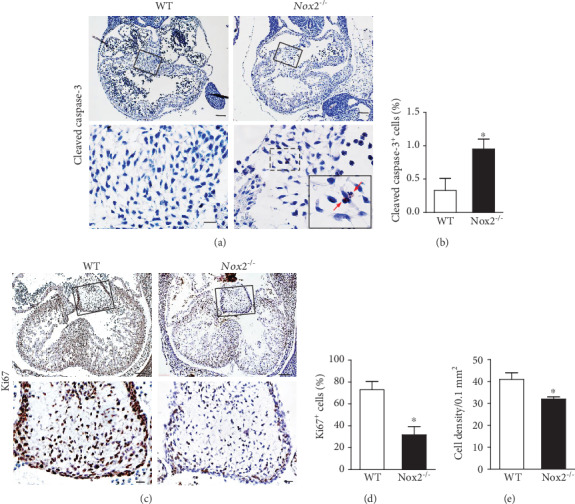
Apoptosis and cell proliferation in endocardial cushions. (a) Representative images of histological sections immunostained for cleaved caspase-3 protein at E10.5. Arrows point to positive cells (brown). (b) Quantification of cleaved caspase-3-positive cells at E10.5. (c) Representative images of Ki67 immunostaining in E12.5 hearts. Quantification of Ki67-positive cells (d) and cell density (e) in endocardial cushions at E12.5. Lower panels (scale bars =10 *μ*m) are enlargement of the boxed areas of the upper panels (scale bars =100 *μ*m) in (a, b). *n* = 5‐7 hearts per group. ^∗^*P* < 0.05 vs. WT by unpaired Student's *t*-test.

ROS regulates cell proliferation and apoptosis in a variety of cell types. Using immunostaining of cleaved caspase-3 protein, we analyzed cell apoptosis in the AV endocardial cushion at E10.5. *Nox2* deficiency resulted in a 2-fold higher apoptosis in the AV endocardial cushion (*P* < 0.05, Figures 7(a) and 7(b)). We also assessed cell proliferation using Ki67 immunostaining (Figure 7(c)). Data was collected from 3-6 heart sections with bulging AV endocardial cushion per heart, a total of 5 hearts per group. Our data show a 50% lower cell proliferation rate in the AV endocardial cushion of *Nox2^−/−^* compared to WT embryos (*P* < 0.001, Figures 7(c) and 7(d)). Furthermore, cellular density in the AV endocardial cushion of *Nox2^−/−^* hearts was significantly lower than that of WT controls (*P* < 0.05, Figure 7(e)).

## 4. Discussion

**Figure 8:**
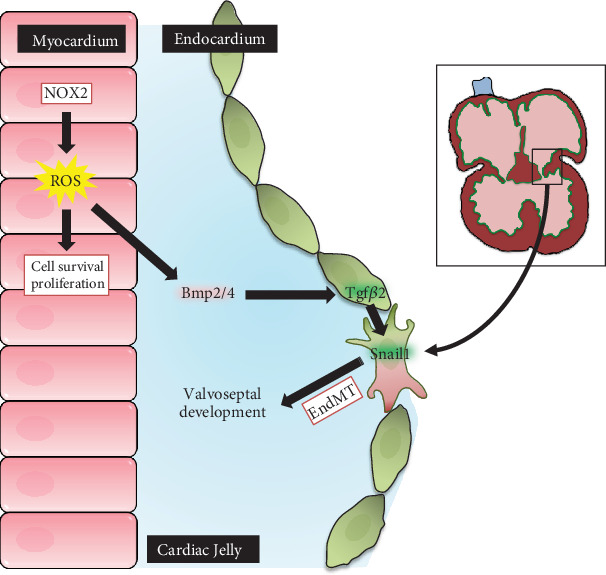
Schematic summary of NOX2-mediated ROS signaling in directing EndMT and valvoseptal development. ROS production from NOX2 positively regulates the expression of factors that promote EndMT and formation of normal valvoseptal structures.

EMT is a process by which epithelial cells undergo their phenotypic transformation to become mesenchymal cells [27, 28]. These multipotent mesenchymal cells are able to differentiate into a variety of cell types. Cells that undergo EMT lose their cell-cell junctions and epithelial cell polarity followed by cytoskeletal reorganization and activation of the mesenchymal gene program. EMT is critical to many developmental, physiological, and pathological processes including organogenesis, wound healing, tissue fibrosis, and cancer metastasis. In heart embryogenesis, EndMT and epicardial EMT are essential for the development of cardiac valves/septum and coronary arteries, respectively [17, 29]. ROS derived from NADPH oxidases have been shown to promote EMT in numerous cell types. For example, ROS-mediated TGF*β* signaling in the regulation of EMT has been shown in keratinocytes [30] and alveolar cells [31]. NOX2-derived ROS signaling has been shown to mediate the EMT process of human breast cancer cells [32]. NOX4 plays a role in TGF*β*-driven EMT in lens epithelial cells to form myofibroblasts, resulting in cataract [33]. ROS signaling is critical to TGF*β*1-induced renal tubular EMT in renal inflammation/fibrosis in angiotensin II-induced hypertension [34]. Additionally, ROS derived from NOX1 and NOX4 have been shown to drive cardiac differentiation and cardiomyocyte proliferation through regulating cardiac transcription factor expression [20, 35, 36]. However, the role of NOX2 in embryonic heart morphogenesis remains unknown. The present study demonstrated that one-third of *Nox2^−/−^* neonates had CHDs including septal defects, AVCD, and AV valve malformation. Importantly, Nox2 deficiency resulted in significantly lower EndMT and cell proliferation in endocardial cushions. Pharmacological inhibition of ROS production impaired EndMT in endocardial cushion explants. Furthermore, *Nox2* deficiency reduced the expression of genes critical to EndMT and AV endocardial cushion development, including *Gata4*, *Tgfβ2*, *Bmp2*, *Bmp4*, and *Snail1*. Our study shows for the first time that NOX2-mediated ROS signaling is critical to AV cushion EndMT, cell proliferation/survival, and normal heart morphogenesis (Figure 8).

A network of signaling molecules and transcription factors in myocardial and endocardial cells regulates EndMT. TGF*β*2 is expressed in the endocardium and myocardium at the AV and OFT regions during cushion formation [37]. BMP2 and BMP4 are released from the myocardium promoting EndMT in the AV canal [25]. Through activation of BMP receptors on endocardial cells, they increase the endocardial expression of *Gata4*, *Tgf-β2*, and *Snail1*, which are essential for EndMT [38]. In the present study, we demonstrated that NOX2 is also expressed in the myocardium overlying the endocardial cushion. The proximity of NOX2 expression allows NOX2-derived ROS to regulate BMP2/4 expression in the myocardium and subsequent TGF*β* signaling in the endocardial cushion. Interestingly, ROS also stimulate Snail1 transcription and EMT in mammary epithelial cells [39, 40]. Additionally, a reduction in ROS signaling from a knockdown of glucose-6-phosphate dehydrogenase, the enzyme that generates NADPH, impairs EMT and embryonic development in zebrafish [41]. The reduced AV cushion EndMT along with decreased expression of *Bmp2/4*, *Tgf-β2*, and *Snail1* in *Nox2^−/−^* hearts in our study strongly suggest that NOX2-derived ROS promotes BMP/TGF*β* signaling and AV cushion EndMT in embryonic heart development.

Cell proliferation and apoptosis are key cellular events that regulate heart development during embryogenesis. It is generally believed that excessive levels of ROS favor cell apoptosis [42]. Consistent with this notion, we recently showed that increased ROS production during pregestational diabetes reduces cell proliferation and increases apoptosis in the endocardial cushion [15]. N-Acetylcysteine treatment restored cell proliferation but not apoptosis. Surprisingly, lowering ROS levels in control mice by N-acetylcysteine treatment increases cell apoptosis [15]. In the present study, deficiency in *Nox2* results in lower ROS in E10.5 hearts, higher cell apoptosis, and lower cell proliferation in the endocardial cushion. These findings suggest that physiological levels of ROS are critical to cell survival and proliferation during embryonic heart development. ROS signaling has been shown to induce developmental gene expression [42]. Thus, lower expression of *Gata4*, *Bmp2/4*, and *Tgf-β2* observed in the present study may lead to higher apoptosis and lower cellular density in the endocardial cushion of *Nox2^−/−^* hearts. The reduced cell proliferation and AV cushion EndMT in combination with higher apoptosis may contribute to CHDs in Nox2^−/−^ mice.

A limitation of this study is the use of a whole body Nox2 knockout mouse. The possible impact of loss of NOX2 expression in tissues outside the cardiovascular system cannot be ruled out. For example, cardiac neural crest cells delaminated from the dorsal neural tube are critical to outflow tract development. It is likely that lack of NOX2 expression in the cardiac neural crest cells may impede their migration to the outflow tract leading to the bicuspid aortic valve, a hypothesis that needs to be further tested in future studies. Additionally, the Nox2^−/−^ mice show a significant but relatively low penetrance of CHDs (34%). It is not clear if other NOX isoforms or ROS-producing enzymes are compensated for the global loss of Nox2 in this model. An extension to this study could be using tissue-specific and inducible animal models or generating animals with deficiency in both Nox2 and 4 genes.

In summary, deficiency in *Nox2* results in congenital defects of the cardiac septum and valves. We further demonstrated that a lack of NOX2-derived ROS production decreases gene expression in the developing heart and disrupts AV cushion EndMT with lower cell proliferation and higher apoptosis in the endocardial cushion. Taken together, our study shows that endogenous ROS signaling from NOX2 is critical to normal heart development in mice. Importantly, our study provides a mechanistic insight into the pathogenesis of congenital heart defects in patients with chronic granulomatous disease [23].

## Figures and Tables

**Table 1:** Sequences of primers used for real-time PCR analysis.

Gene	Accession No.	Product size (bp)	Primer sequence (5′ → 3′)
Bmp2	NM_007553.3	151	F: CAAACACAAACAGCGGAAGC R: CAGCAAGGGCAAAAGGACAC
Bmp4	NM_007554.2	250	F: GTTATGAAGCCCCCAGCAGA R: CCCAATCTCCACTCCCTTGA
Gata4	NM_008092.3	134	F: GCCTGCGATGTCTGAGTGAC R: CACTATGGGCACAGCAGCTC
Gata5	NM_008093.2	167	F: ACCCCACAACCTACCCAGCA R: GCCCTCACCAGGGAACTCCT
Mef2c	NM_001170537.1	405	F: CACCGAGTACAACGAGCCGCA R: CTGGTGCCTGCACCGGATGTC
Nkx2.5	NM_008700.2	162	F: GACAGCGGCAGGACCAGACT R: CGTTGTAGCCATAGGCATTG
Snail1	NM_011427.2	114	F: CACACGCTGCCTTGTGTCT R: GGTCAGCAAAAGCACGGTT
Tbx5	NM_011537.3	103	F: AGGAGCACAGTGAGGCACAA R: GGGCCAGAGACACCATTCTC
Tgf-*β*2	NM_009367.3	230	F: CTGTGCAGGAGTGGCTTCAC R: GCAGGAGATGTGGGGTCTTC
Notch1	NM_008714.3	142	F: CAGCTTGCACAACCAGACAGA R: TAACGGAGTACGGCCCATGT
28S	NR_003279.1	178	F: GGGCCACTTTTGGTAAGCAG R: TTGATTCGGCAGGTGAGTTG

F and R indicate forward and reverse primers, respectively.

**Table 2:** Incidence of congenital heart defects in *Nox2^−/−^* mice at P0.

	Wild type (*N* = 35 of 5 litters)		*Nox2^−/−^* (*N* = 61 of 12 litters)	
	*N*	%	*N*	%
Normal	35	100	40	65.6^∗∗^
Abnormal	0	0	21	34.4^∗∗^
ASD	0	0	11	18^∗^
VSD	0	0	11	18^∗^
AVCD	0	0	2	3.3
BAV	0	0	4	6.6

ASD: atrial septal defect; VSD: ventricular septal defect; AVCD: atrioventricular canal defect. ^∗^*P* < 0.05, ^∗∗^*P* < 0.01 vs. wild-type by the Chi-squared test.

## Data Availability

The data that support the findings of this study are available from the corresponding author upon reasonable request.
